# Baseflow significantly contributes to river floods in Peninsular India

**DOI:** 10.1038/s41598-024-51850-w

**Published:** 2024-01-13

**Authors:** Shailza Sharma, P. P. Mujumdar

**Affiliations:** 1 Department of Civil Engineering, Indian Institute of Science, Bangalore, India; 2 Interdisciplinary Centre for Water Research, Indian Institute of Science, Bangalore, India

**Keywords:** Environmental sciences, Hydrology

## Abstract

Extreme rainfall prior to a flood event is often a necessary condition for its occurrence; however, rainfall alone is not always an indicator of flood severity. Antecedent wetness condition of a catchment is another important factor which strongly influences the flood magnitudes. The key role of soil moisture in driving floods is widely recognized; however, antecedent conditions of deeper saturated zone may contribute to river floods. Here, we assess how closely the flood magnitudes are associated to extreme rainfall, soil moisture and baseflow in 70 catchments of Peninsular India for the period 1979–2018. Annual flood magnitudes have declined across most of the catchments. Effect of flow regulations is also assessed to understand the impact of human interventions on flood characteristics. Reservoir regulation has positive effect by reducing the flood peak and volume, whereas the duration of flood events has increased after the construction of dams. Baseflow exhibits similar patterns of trends as floods, whereas trends in rainfall and soil moisture extremes are weakly correlated with trends in flood magnitudes. Baseflow is found to be more strongly influencing the flood magnitudes than soil moisture at various time lags. Further analysis with event coincidence analysis confirms that baseflow has stronger triggering effect on river floods in Peninsular India.

## Introduction

Understanding the changing characteristics of floods^1^ and their relationship with the physical causative mechanisms is a prerequisite for developing effective flood management strategies^2,3^. Physical causes of short term variability and long term changes in extreme floods vary between the catchments^4,5^. Investigating the relative importance of key drivers of floods is therefore critical for improving the scientific understanding of catchment dynamics. Changing characteristics and causes of floods are well documented across many catchments in Europe^3,6–10^ and United States^2^ with extreme rainfall, soil moisture excess and snowmelt as potential drivers. Influence of rainfall and soil moisture extremes on flood peaks is evaluated in Australian^11,12^ and African^13^ catchments. However, there is no systematic study on identifying the importance of flood generating mechanisms in Indian catchments.

There is significant evidence that rainfall extremes are intensifying in response to warming^14,15^, whereas the evidence for increase in floods remains elusive^16^. Increasing trends in rainfall extremes^17–20^ and increase in the flood risk^21,22^ are reported in India. However, studies on understanding the physical causes of such trends remain limited^23–25^. A recent study identifies multiday rainfall as a prominent driver of floods in India by examining the soil moisture conditions and rainfall before high flow events simulated using the Variable Infiltration Capacity (VIC) model^26^. Authors adopt an event-based approach to identify the flood drivers but the analysis does not consider the role of groundwater in triggering floods. Floods have serious impacts on agriculture, infrastructure, water resources systems and reservoir operations. Therefore, a detailed assessment is required to classify the flood generating mechanisms in Indian catchments. Identifying the hydrological processes which trigger floods will not only improve our understanding of flood mechanisms in Indian catchments but it will also provide a foundation for robust flood risk assessment.

Rainfall and subsurface antecedent wetness conditions prior to the flood event are the primary drivers of floods in India^24,26^ as snowmelt triggers floods in a few catchments^27,28^. The role of soil moisture in driving river floods is widely recognized in literature^2,3,11–13^, whereas groundwater is not considered in flood related studies. Groundwater plays an important role in maintaining the flow of rivers, but its influence on floods is poorly understood^29^. Groundwater well observations are sparse and may not represent the influence of water storage in deeper saturated zone on floods. Therefore, baseflow is used to understand the role of groundwater storage in controlling floods in Peninsular India.

Annual maximum flows are the largest floods experienced in a year and often represent the most disastrous flood event. Trends in the annual flood magnitudes are estimated to understand the changes in water availability. Impact of reservoirs is also examined in this study to understand the influence of flow regulations in Peninsular India. The natural flow regime of rivers is largely altered due to boom in dam construction across the world during the last century^30^. The regulation of rivers with reservoirs for different purposes such as irrigation, hydropower, water supply and flood control significantly alters the downstream flow by storing and releasing water with certain operation rules. Flow regulations affect the magnitude, frequency and timing of downstream high and low flows^31–34^. Therefore, it is important to study the impact of reservoirs on the flow regime using downstream streamflow records. This study presents the analysis of pre- and post-dam construction high flow changes to understand the influence of reservoirs on annual flood characteristics: peak, volume and duration. Understanding the extent to which the reservoir regulations affected the flood characteristics is crucial for designing better reservoir operation rules in Peninsular catchments.

The importance of rainfall, soil moisture and baseflow for generating floods in Peninsular India is investigated first using Kendall’s rank correlation coefficient and Pearson correlation coefficient for different lags at annual timescale. However, high flows with a little lower magnitude than the annual maximum flow can occur in the same year and in the same catchment. In addition, drivers of these floods of different magnitudes can be quite different from annual maximum floods. Event-based approach which extracts the sample using peaks-over-threshold is more robust to assess the importance of flood generating mechanisms^13,26^. Therefore, further investigation is performed using Event Coincidence Analysis (ECA), which tests for possible causal influence of flood drivers in triggering the flood events of little lower magnitude than annual floods. Triggering effect is evaluated using the statistics based on trigger coincidences for the condition that extreme rainfall, soil moisture and baseflow are followed by the flood events. ECA results are used to find the dominant driver which has a higher influence on the flood events in Peninsular catchments.

## Study area

**Figure 1:**
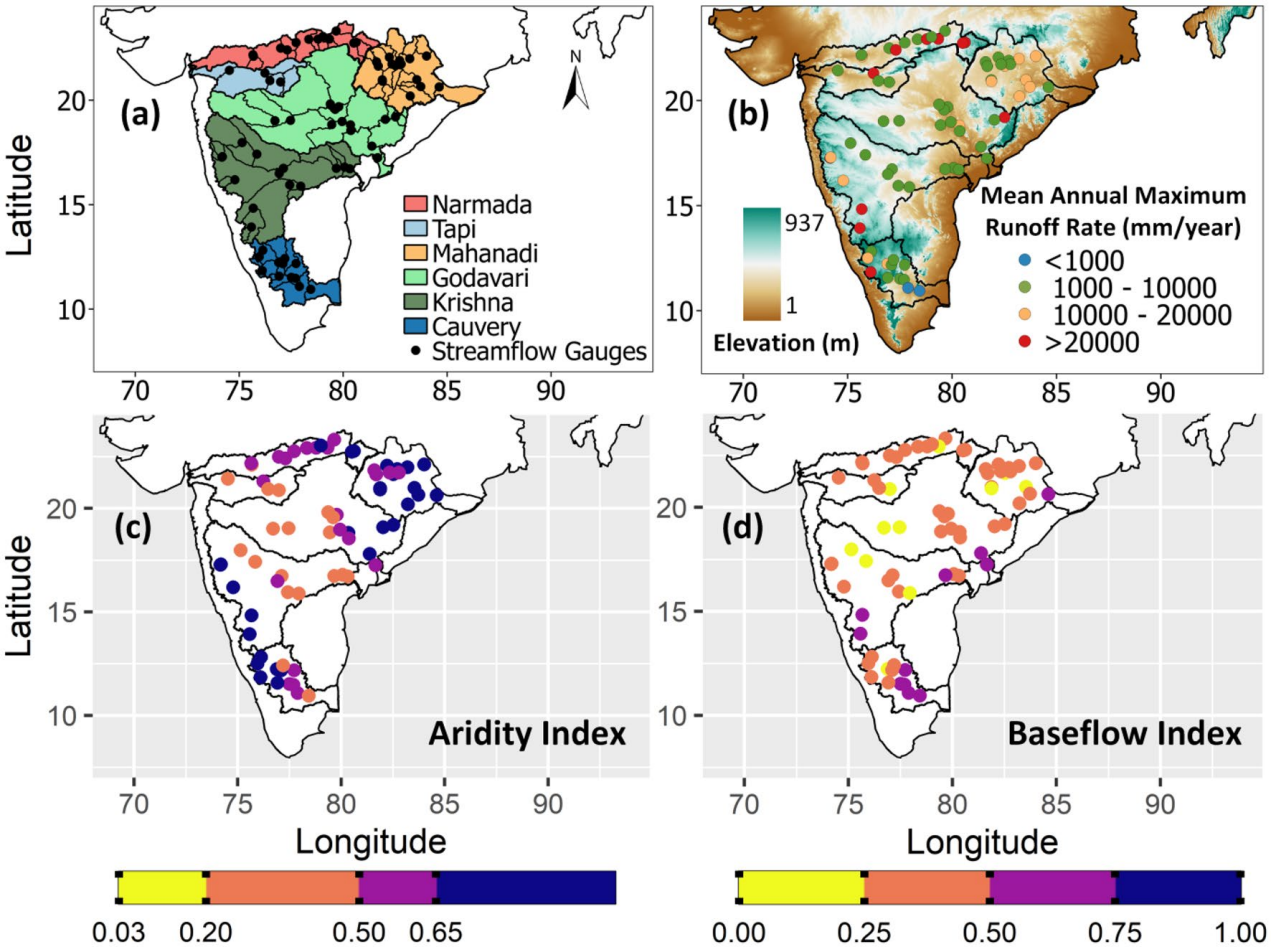
(**a**) Locations of streamflow gauges with catchment boundaries in six major river basins of Peninsular India, (**b**) elevation map and mean annual maximum runoff rate (streamflow per unit catchment area), (**c**) Aridity Index (AI) and (**d**) Baseflow Index (BFI). The maps in first row are prepared in QGIS (Version 2.14.0 ‘Essen’ (2016), URL: http://qgis.org) and the maps in second row are generated using R (Version 4.2.2 (2022), URL: https://www.R-project.org/).

Narmada, Tapi, Mahanadi, Godavari, Krishna and Cauvery are six major river basins of Peninsular India. Tapi is the smallest river basin with an area of 65,145 km^2^ and Godavari is the largest which covers an area of 312,812 km^2^. Narmada and Tapi are west flowing rivers which join the Arabian Sea, while other four are east flowing rivers which drain into the Bay of Bengal. Godavari is the longest river of length 1465 km and Cauvery is the shortest river with a length of 560 km in Peninsular India. The locations of 70 selected catchments are shown in Fig. 1a. The catchment areas vary in size from 1260 to 307,800 km^2^ (Fig. S1). The elevation varies between a minimum of 1 m to a maximum of 937 m (Fig. 1b). Spatial variation of mean annual maximum runoff rate averaged over a period of 40 years is shown in Fig. 1b. High runoff rates are observed in Narmada, lower and middle Mahanadi, Krishna upper sub-basin, Tungabhadra upper sub-basin and Cauvery upper sub-basin. Aridity Index (AI) defined as the ratio of mean annual precipitation to mean annual potential evapotranspiration is shown in Fig. 1c. United Nations Environment Programme (UNEP)^35^ provides a climate classification scheme based on the Aridity Index values. Peninsular catchments have semi-arid (AI 0.2–0.5), dry sub-humid (AI 0.5–0.65) and humid (AI > 0.65) climate conditions. Spatial variation of Baseflow Index (BFI), the long-term ratio between baseflow to total streamflow is shown in Fig. 1d. The distribution of BFI is relatively even with BFI values between 0.25 and 0.50 for most of the catchments.

## Results

### Trend analysis

**Figure 2:**
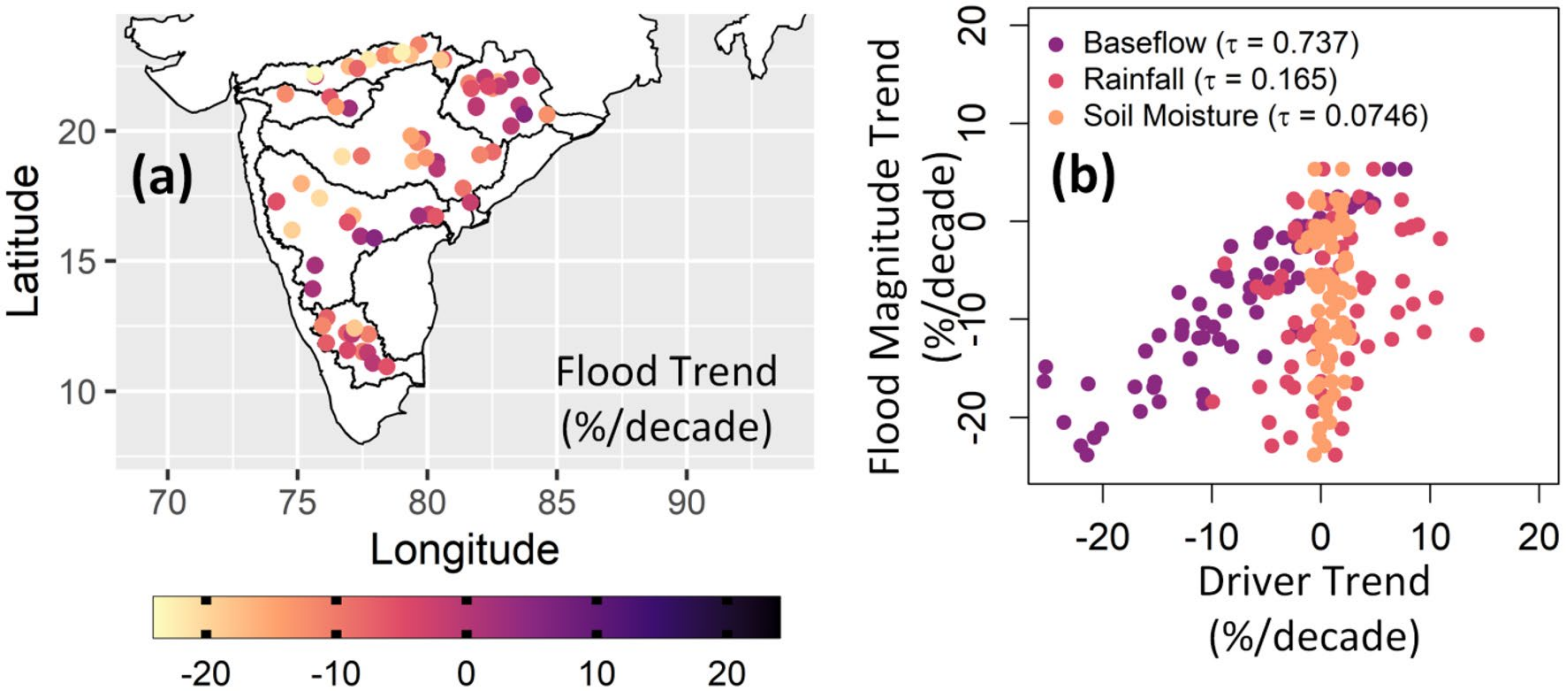
(**a**) Trends in annual flood magnitudes and (**b**) association of trends in flood magnitudes with trends in flood drivers. Flood magnitudes are shrinking across most of the catchments in Peninsular India. Trends in floods are strongly correlated with trends in baseflow (Kendall’s $\tau =0.737$) and weakly correlated with soil moisture (Kendall’s $\tau =0.0746)$. The figure is prepared in R (Version 4.2.2 (2022), URL: https://www.R-project.org/).

Annual flood magnitudes have decreased in Peninsular catchments over the period 1979–2018 (Fig. 2a). An increase in flood magnitudes is observed only in two catchments (Kurubhata and Kantamal) of Mahanadi river basin and one catchment (Dameracherla) of Godavari river basin. Flood magnitudes are declining drastically in Narmada river basin. Trends in flood magnitudes show a strong association with trends in annual mean baseflow (Fig. 2b). Signs of flood magnitude trends are more consistent with the signs of trends in baseflow compared to rainfall and soil moisture. The strength of dependence between trends in flood magnitudes and trends in flood drivers is summarized with Kendall rank correlation coefficient. A high value of Kendall’s $\tau =0.737$ is observed for the pairs of trends in annual flood magnitude and trends in mean annual baseflow across all the Peninsular catchments. Trends in annual maximum daily rainfall ($\tau =0.165$) and annual mean soil moisture ($\tau =0.0746$) show a weak correlation with trends in flood magnitudes. These results suggest that floods in Peninsular catchments are strongly correlated with baseflow compared to rainfall and soil moisture.

### Effect of flow regulations on flood characteristics

**Figure 3:**
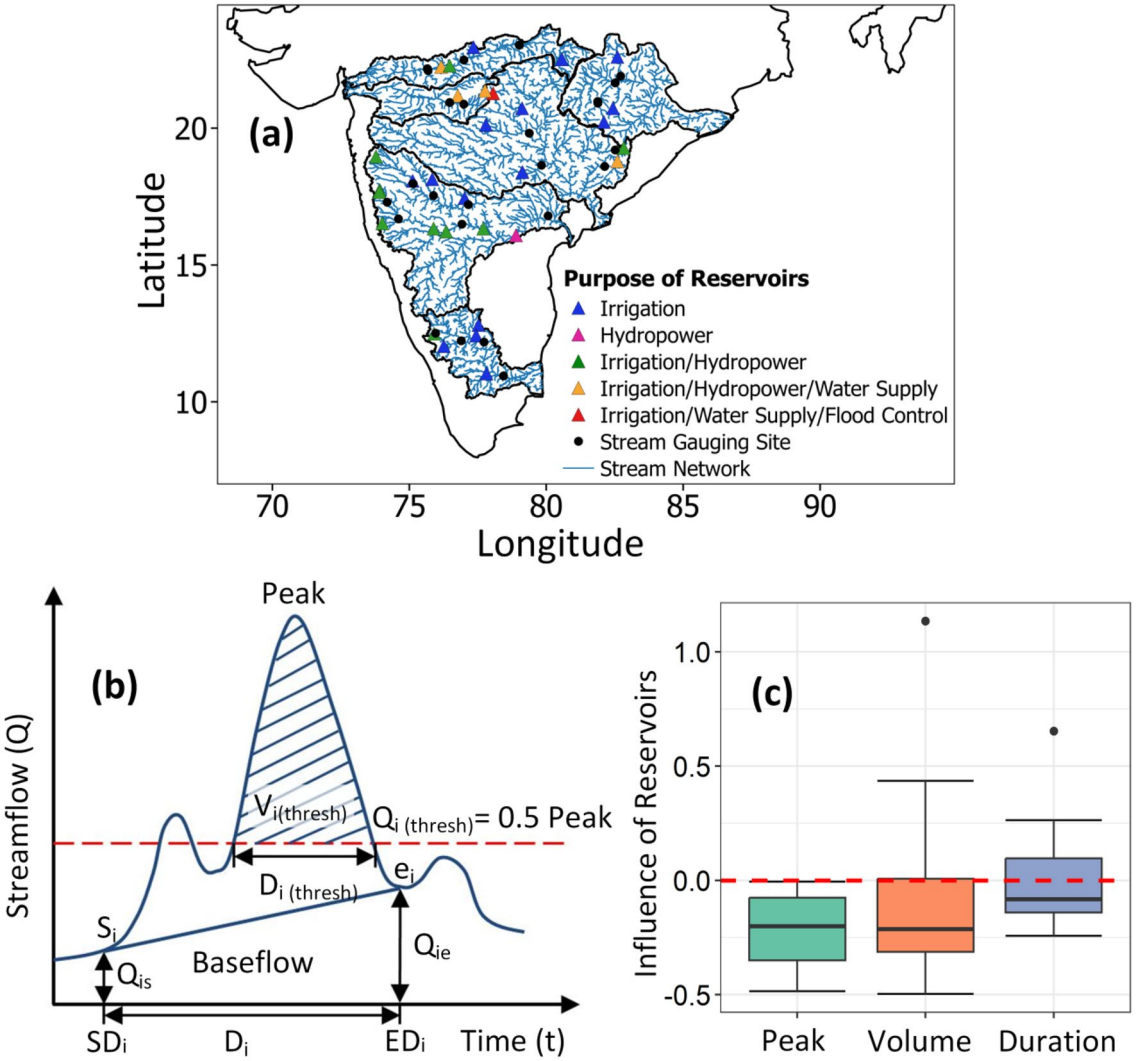
(**a**) Locations of reservoirs and streamflow gauges lying downstream on the same flow lines, (**b**) schematic diagram to derive the flood characteristics and (**c**) effect of flow regulations on flood peak, volume and duration. Floods last longer but became less severe with reduced peak and volume after the construction of dams. The map in first row is prepared in QGIS (Version 2.14.0 ‘Essen’ (2016), URL: http://qgis.org), (**b**) is generated using Microsoft Word (Version 2010 https://www.office.com/), and (**c**) is prepared in R (Version 4.2.2 (2022), URL: https://www.R-project.org/).

The impacts of reservoir flow regulations in Peninsular catchments are assessed using “*pre-post-disturbance*” approach in this study. Locations of dams are marked on the streamflow network and the streamflow gauges downstream of these dams on the same flow lines are identified (Fig. 3a). Total 31 dams are considered which came after 1980 as per the information from National Register of Large Dams (NRLD) and 25 streamflow gauges are marked which have a good length of flow records available for pre-disturbance period. When there is more than one reservoir upstream of a stream gauge, impact is evaluated for the structure which came first and year of construction decides the length of records for the new dam downstream of the old dam. The pre-dam construction period of new dam begins from the year of construction of the older dam lying upstream and ends at its own year of construction. Annual flood peak, volume and duration are computed for pre- and post-dam construction period as illustrated in Fig. 3b. The comparison of mean flood characteristics for the two periods shows that reservoir regulations have strong influence on flood characteristics. Reservoir regulation has increased the flood duration by up to 65% while it has reduced the peak flow and flood volume by ~ 48.5% and ~ 50%, respectively. Floods after the construction of dams last longer but are less severe with reduced peak and volume in Peninsular catchments. These impacts are independent of the purpose of reservoirs. Upper Wardha dam which serves the purpose of flood control along with irrigation and water supply shows reduction in all the flood characteristics (peak − 21.5%, volume − 32.1% and duration − 14%). Flood alleviation effect of reservoirs is observed in different parts of the world^31,34,36^. Reduction in flood severity indicates a positive effect of dam construction on flood alleviation in Peninsular India.

### Importance of flood drivers

**Figure 4:**
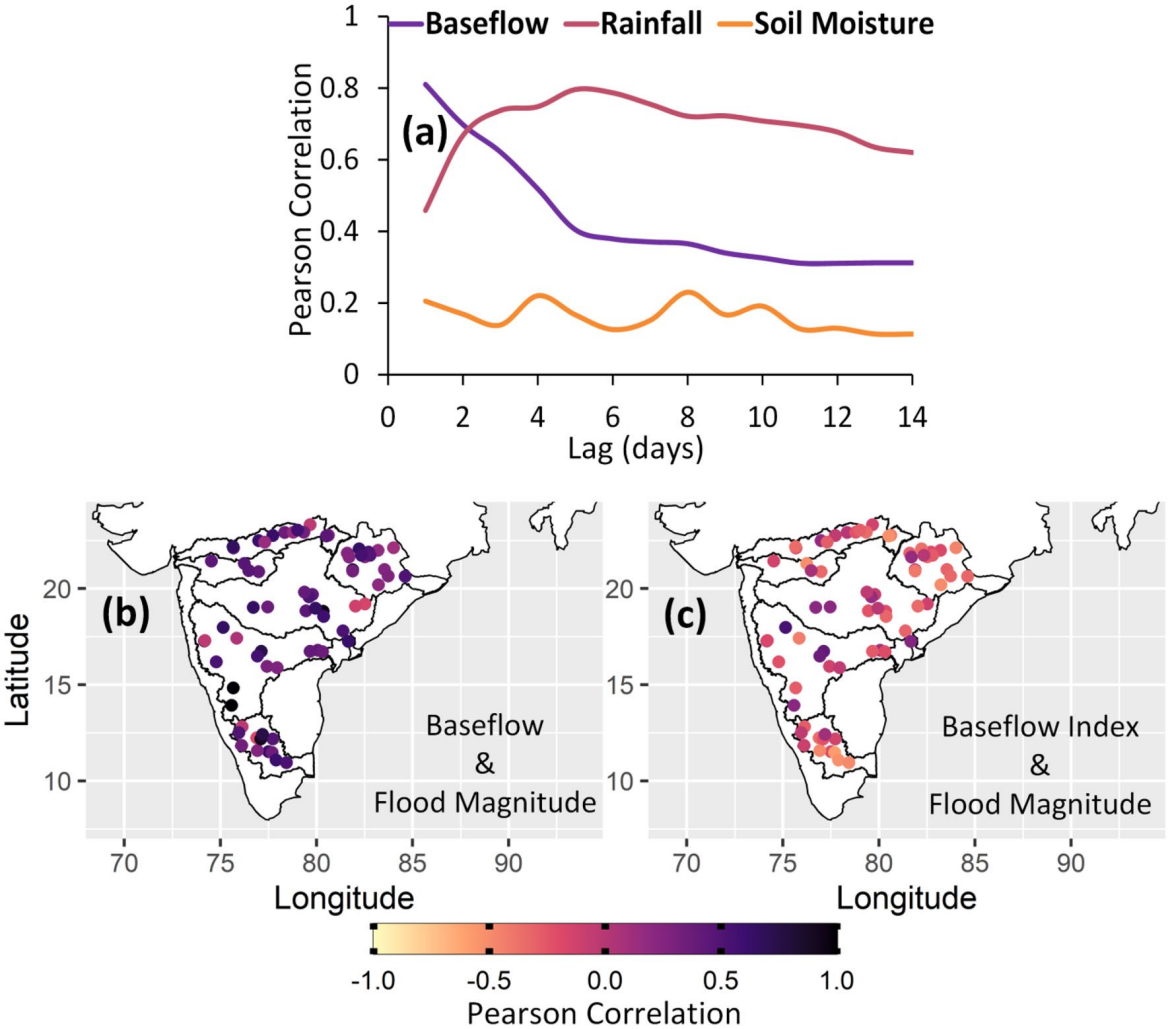
(**a**) Correlation between flood magnitude and flood drives (baseflow, rainfall and soil moisture) for a range of antecedent periods (1–14 days) in a randomly selected Peninsular catchment. Baseflow has a stronger and long lasting association with flood peaks than soil moisture at annual time scale. (**b**) Spatial variation of correlation between flood magnitude and baseflow 5 days before the flood event. (**c**) Spatial variation of correlation between flood magnitude and baseflow index on flooding day. All parts of the figure are generated using R (Version 4.2.2 (2022), URL: https://www.R-project.org/).

It is well established in literature that antecedent soil moisture conditions play an important role in the hydrological response of a catchment^11,12,16,37^. However, importance of antecedent conditions may extend deeper into the saturated zone as revealed in a recent study by Berghuijs and Slater^29^. Here, we investigated the association of annual floods and flood drivers (baseflow, rainfall and soil moisture) with Pearson correlation coefficient for a range of antecedent periods from 1 to 14 days (Fig. 4a). Instantaneous values of baseflow and soil moisture are used, whereas accumulated rainfall from a specific lag to the flooding day are used in the correlation analysis. Baseflow shows a strong correlation with annual maximum flows at all the time lags compared to soil moisture. Baseflow dominates for the first few lags i.e. less than 3–4 days and rainfall dominates at longer antecedent periods for 50 catchments. For remaining 20 catchments (especially from Cauvery river basin), baseflow dominates for more than 5–7 days and rainfall dominates for more longer antecedent periods as shown in Fig. S2. A catchment with higher baseflow reflects more wet conditions, which means the chances of rapid runoff are high with the incoming rainfall event. On the other hand, the correlation between accumulated rainfall and flow peaks is relatively high at longer time lags because rainfall not only drives the flood peak but it also contributes to soil moisture and groundwater levels. Accumulated rainfall over a longer period will eventually raise the baseflows and thus more water will be contributed to river flows. Results are shown for a randomly selected catchment Bamini with semi-arid climate in Godavari river basin. Similar correlation pattern is observed across 50 catchments of Peninsular India. At short time scales, flood magnitudes are strongly associated to baseflow than rainfall and soil moisture. This observation suggests that baseflow contributes more water to the Peninsular catchments.

The spatial pattern of correlation between flood magnitudes and baseflow computed 5 days before the flood events is shown in Fig. 4b. High positive correlation is observed across all the catchments indicating a strong association of baseflow with floods. This highlights the strong influence of baseflow on floods in Peninsular catchments. Relative contribution of baseflow to peak flows is further investigated using Baseflow Index (BFI) on the flooding day. This will help in quantifying the fraction of peak flow which comes from baseflow. Negative correlation is observed between flood magnitudes and BFI (Fig. 4c). This suggests that although baseflow contributes more to river flows (Fig. 4b); however, its contribution to the event flow magnitude decreases as surface runoff contributes a higher fraction of flood discharge than baseflow. Additionally, a flood event cannot occur without high rainfall even if the landscape has higher baseflow.

**Figure 5:**
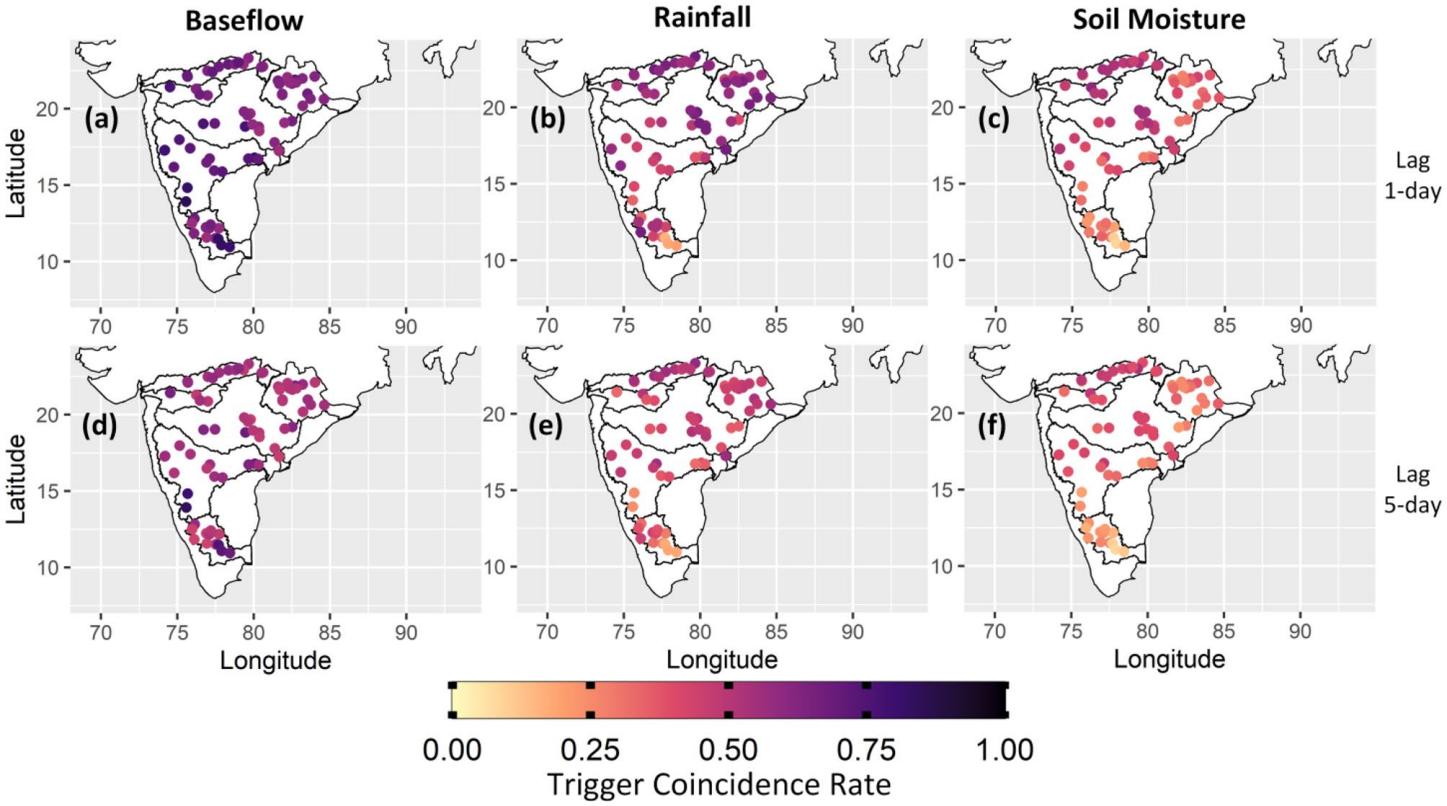
Triggering effect of baseflow, rainfall and soil moisture on floods defined above 95th percentile threshold. Baseflow and soil moisture are based on their instantaneous values before the flood event; whereas rainfall is the 5 days accumulated rainfall. Tigger coincidence rates are high for baseflow 1 day (first row) and 5 days (second row) prior to the flood event. Baseflow has stronger triggering effect on floods in Peninsular India. The maps are prepared in R (Version 4.2.2 (2022), URL: https://www.R-project.org/).

The importance of flood drivers is evaluated using the trigger coincidence rate for the period 1979–2018. The p-values are lower than the confidence level $\alpha =0.01$, therefore the null hypothesis of independent random series is rejected for all the catchments. All the trigger coincidence rates are statistically significant. Trigger coincidence rates are high for baseflow compared to rainfall and soil moisture across all the Peninsular catchments (Fig. 5). Baseflow 1 day prior to the flood event is significantly influencing the river floods, whereas soil moisture on the previous day has lowest triggering effect on floods (Fig. 5a–c). This suggests that high baseflow conditions are coinciding more with the severe 95th percentile flood events. The triggering effect of baseflow is longer-lasting as the trigger coincidences remain high compared to other two flood drivers at a time lag of 5 days (Fig. 5d–f). Baseflow on the previous day of flood has a higher trigeering effect than longer time lag. ECA results corroborate the statement that baseflow significantly contributes to floods in Peninsular India.

The role of flood drivers may change with time; therefore, investigation is carried out for two periods to better understand the evolving nature of floods and their association with flood drivers. Data is divided into two equal halves from 1979–1998 to 1999–2018. Triggering effect of flood drivers is assessed for 85th as well as 95th percentile extremes to understand the influence on floods of different magnitudes (Figs. S3–S4). Baseflow has high trigger coincidence rates compared to rainfall and soil moisture irrespective of the flood magnitude and record length. These findings suggest that baseflow plays a critical role in controlling floods in Peninsular India and future floods depend on the pre-existing baseflow conditions during high rainfall events.

## Conclusions

The active role of groundwater in storm runoff in streams is discovered decades ago^38,39^. Baseflow also exerts significant influence over the entire flood frequency curves^40^. Despite the significant role of groundwater in storm runoff generation, groundwater is rarely considered in flood related studies. Recent studies consider the critical role of soil moisture in modulating floods^2,3,11–13^, whereas groundwater is often overlooked. Present study extends our knowledge on process based controls on floods. Our analysis reveals that pre-existing baseflow conditions play an important role in driving floods. Baseflow is the dominant driver of floods at shorter time lags and rainfall controls flood magnitudes at longer antecedent periods. The effect of baseflow is stronger than soil moisture and lasts for longer antecedent periods in Peninsular catchments.

Presence of reservoir in a catchment significantly influences the natural flow regime by storages and releases. Reservoir regulation has reduced flood severity i.e. flood peak and flood volume but duration of flood events has increased after the construction of dams in Peninsular catchments. This attenuation in flood severity is independent of the purpose of reservoirs. A reduction in flood peak and volume is achieved due to the retention of water in the reservoir and by releasing this excess water over longer durations. Reservoir regulation has positive effect by alleviating the flood severity in Peninsular India.

One potential limitation of present study is that we identified single dominant mechanism of floods in the catchments. However, floods in a catchment can arise through a combination of different mechanisms. Present analysis can be further extended by conditioning on the combination of flood drivers using multivariate statistical tools to accurately estimate their combined effect on river flooding. Incorporating more information on the flood generating mechanisms is the key for improving flood predictions and plan better preventive measures.

## Methods

### Datasets

Daily streamflow time series for 70 catchments in six major river basins of Peninsular India (Fig. 1a) are obtained from India Water Resources Information System (https://indiawris.gov.in/wris/). Gaps in the daily streamflow records are filled using time series methods for synthesizing missing streamflow records^41,42^. Daily high resolution rainfall^43^ dataset on a grid size of 0.25° is obtained from India Meteorological Department (IMD). European Space Agency Climate Change Initiative (ESA CCI) soil moisture^44–46^ with daily temporal and 0.25° spatial resolution is used in this study. High resolution Aridity index values are obtained from Global Aridity Index and Potential Evapotranspiration Database—Version 3 (Global-AI_PET_v3)^47^. Information on location, year of construction, capacity and purpose of reservoirs is collected from Central Water Commission (CWC) report on National Register of Large Dams^48^. Catchments are delineated in Quantum Geographic Information System (QGIS) using Digital Elevation Model (DEM) obtained from Shuttle Radar Topographic Mission (SRTM) at 30 m spatial resolution (https://srtm.csi.cgiar.org/srtmdata/). Catchment average rainfall, soil moisture and aridity index are calculated across the selected catchments of Peninsular India. ESA CCI daily soil moisture (COMBINED) data product is available from 1978; therefore a common period of 40 years (1979–2018) is selected based on the availability of data for all the variables.

### Baseflow separation

George and Sekhar^49^ finds Ekhardt filter^50^ more suitable compared to other digital filters for baseflow separation in Kabini basin, a tributary of Cauvery river in Western Ghats, India. Therefore, Ekhardt filter, a two-parameter recursive filter is used to estimate baseflow in the study area. The filter equation is given by

1 $$b_{i} = \frac{{\left( {1 - BFI_{\max } } \right)\alpha b_{i - 1} + \left( {1 - \alpha } \right)BFI_{\max } Q_{i} }}{{1 - \alpha BFI_{\max } }}$$

where α is the recession constant and ${BFI}_{{\text{max}}}$ is the maximum baseflow index modelled by the algorithm, ${b}_{i}$ is the baseflow, and ${Q}_{i}$ is discharge for time step $i$. Peninsular catchments are underlain by hard rock aquifers; therefore ${BFI}_{{\text{max}}}=0.25$ is selected. Recession constant is computed based on the master recession curve (MRC) method described in WMO manual on low-flow estimation and prediction^51^. The beginning of recession is marked below the ${Q}_{70}$ threshold at least two days after the peak flood discharge. Segment length is computed for each catchment and MRC is obtained by plotting pairs of ${Q}_{t-1}$ and ${Q}_{t}$. Recession constant α is estimated as the slope of the curve. The procedure is illustrated for a randomly selected Haralahalli catchment of Krishna river basin in Fig. S5.

### Trend estimation

Trends in annual maximum streamflow are detected using Mann–Kendall trend test^52^ and the slope of linear trends in Peninsular catchments is computed using Sen-Theil slope estimator^53^. In order to facilitate a relative comparison of trends across catchments of different sizes, they are expressed in units of percentage change per decade following previous studies^54–56^, such that

2 $$T = \frac{S \times 10\;years}{{\overline{x}}} \times 100$$

where $T$ is the trend in %/decade, $S$ is the Sen’s slope and $\overline{x }$ is the mean of annual maximum streamflow time series. Decadal trends in the flood drivers are estimated similarly.

### Extracting flood characteristics

Annual flood peak, volume and duration series are extracted using the procedure followed in previous studies^57–60^. Annual maxima of streamflow data is the peak flow. Baseflow is used as the criterion to delineate flood hydrograph and derive flood volume and flood duration. Start day of flood runoff is marked as the abrupt rise in discharge (above the baseflow) and flattening of the recession limb (a return to baseflow) is the end day as shown in Fig. 3b. Flood duration for the selected year $i$ is ${D}_{i}=\left(E{D}_{i}-S{D}_{i}\right).$ Flood volume for *i*th year with observed streamflow ${Q}_{ij}$ on *j*th day is computed as follows

3 $$\begin{gathered} V_{i} = \left( {V_{i}^{Total} - V_{i}^{Base} } \right) \hfill \\ \quad \;\; = \mathop \sum \limits_{{j = SD_{i} }}^{{ED_{i} }} \left\{ {Q_{ij} - \frac{1}{2}\left( {Q_{is} + Q_{ie} } \right)} \right\} - \frac{1}{2}D_{i} \left( {Q_{is} + Q_{ie} } \right) \hfill \\ \end{gathered}$$

where ${Q}_{is}$ and ${Q}_{ie}$ are the observed daily streamflows on the start and end dates of flood runoff, respectively. In this study, a flood event is defined as the upper part of the hydrograph lying above the fixed threshold ${Q}_{i\left(thresh\right)}=0.5\times Peak Flow$ as described by Karmakar and Simonovic^59^. Flood duration is ${D}_{i\left(thresh\right)}$ and flood volume ${V}_{i\left(thresh\right)}$ is estimated for the threshold discharge after deducting the baseflow volume.

### Quantifying the impact of flow regulations

Human activities like construction of reservoirs significantly affect the hydrological system by disturbing the natural flow conditions. Paired-catchment approach is the classical method in catchment hydrology to detect the impact of a disturbance on the flow regime^34,61,62^. The flow regimes of two nearby catchments with similar physical characteristics are compared in this method by setting one as a benchmark catchment and other as a disturbed catchment. Indian catchments are bigger in size and it is difficult to find adequate number of pairs with the presence of a large number of hydraulic structures in a single catchment. Therefore, the “*pre-post-disturbance*” approach which compares hydrologic extremes before and after a disturbance is used in this study to quantify the impact of human influence. A minimum of 15 years and an optimum of 20 years for each part are required such that the normal, dry and wet years within each period are equally distributed^63,64^. Stream networks are delineated for the Peninsular river basins and locations of dams are marked on the network. The streamflow gauging stations which lie downstream of the dams on same flowlines are identified. A comprehensive analysis on changes in flood characteristics (peak, volume and duration) is conducted by dividing the streamflow records into two parts: the undisturbed period and the disturbed period. Length of streamflow records varies between a maximum of 52 years (1967–2018) to a minimum of 40 years (1979–2018) for quantifying the changes in flood characteristics. For a robust assessment of changes only the structures constructed after 1980 are considered so that a good length of records is available before a disturbance. Changes in the flood characteristics are estimated as $({C}_{D}-{C}_{U})/{C}_{U}$, where ${C}_{D}$ and ${C}_{U}$ are the mean characteristics after disturbance and before the disturbance, respectively.

### Event coincidence analysis

Event Coincidence Analysis (ECA) is a recently developed statistical tool exclusively designed for measuring the strength, directionality and time lag of statistical interdependency between two event series^65,66^. Donges et al.^67^ used the ECA framework to investigate the role of floods as triggers of epidemic outbreaks with country-level observational data. Manoj et al.^68^ employed ECA to identify and quantify the preconditioning of precipitation extremes by soil moisture anomalies over India. ECA is suitable to test for existence, direction and significance of possible relationship between pairs of two event series^68,69^ $X$ and $Y$. ECA is utilized in this study to test for existence and significance of statistical interrelationship of floods with the flood drivers.

Let $X$ be flood events occurring at timings $\left\{{t}_{1}^{X},\dots ,{t}_{{N}_{X}}^{X}\right\}$ and $Y$ be the flood drivers (rainfall, soil moisture and baseflow) occurring at times $\left\{{t}_{1}^{Y},\dots ,{t}_{{N}_{Y}}^{Y}\right\}.$ ${N}_{X}$ and ${N}_{Y}$ are the number of events of event series $X$ and $Y,$ respectively. The event series are assumed to cover a time interval $\left({t}_{0},{t}_{e}\right)$ with length $T={t}_{e}-{t}_{0}$, such that $t_{0} \le t_{1}^{X} \le \cdots \le t_{{N_{X} }}^{X} \le t_{e}$ and $t_{0} \le t_{1}^{Y} \le \cdots \le t_{{N_{Y} }}^{Y} \le t_{e}$ which yields the event rates ${\lambda }_{X}={N}_{X}/T$ and ${\lambda }_{Y}={N}_{Y}/T.$ Coincidences of events in both the series are counted and the strength of statistical interrelationship is quantified using a measure called “*Trigger Coincidence Rate*” ${(r}_{t})$. It measures the fraction of $Y$-type events that are followed by at least one $X$-type event. Multiple $X$-type events within the coincidence interval are counted only once.

Trigger coincidence rate^67^ is defined as

4 $$r_{t} \left( {\Delta T,\tau } \right) = \frac{1}{{N_{Y} }}\mathop \sum \limits_{j = 1}^{{N_{Y} }} F\left[ {\mathop \sum \limits_{i = 1}^{{N_{X} }} I_{{\left[ {0,\Delta T} \right]}} \left( {\left( {t_{i}^{X} - \tau } \right) - t_{j}^{Y} } \right)} \right]$$

where $\Delta T$ is the coincidence interval and $\tau$ is the time lag parameter. An instantaneous coincidence occurs if events of two event series occur closer in time i.e. if the condition ${t}_{i}^{X}-{t}_{j}^{Y}\le \Delta T$ is satisfied. A lagged coincidence occurs when the $X$ events shifted by time lag $\tau$ i.e. at time ${(t}_{i}^{X}-\tau )$ coincide with the $Y$-type event and the condition ${(t}_{i}^{X}-{\tau )-t}_{j}^{Y}\le \Delta T$ holds. $F\left(\cdot \right)$ denotes the Heaviside function which conveys information on whether the flood drivers $(Y)$ have a triggering effect on flood $(X)$ events or not. The values of ${r}_{t}$ vary between 0 (complete absence of triggering effect between $X$ and $Y$) and 1 ($X$ events succeed all the $Y$ events).

### Testing the significance of coincidences

The two event series are assumed to be randomly distributed and mutually independent over the continuous time interval $T$. The occurrences of coincidences are rare and thus $X$ and $Y$ event time series are treated as two independent Poisson processes. This allows derivation of distributions of coincidence rates to test the statistical significance of ECA results. The probability of occurrence of a given number of trigger coincidences $(K={N}_{X}{\cdot r}_{t})$ between two event series can be approximated by Binomial distribution^66^

5 $$P\left( {K;N_{Y} , 1 - \left( {1 - p} \right)^{{N_{X} }} } \right) = \left( {\begin{array}{*{20}c} {N_{Y} } \\ K \\ \end{array} } \right)\left( {1 - \left( {1 - \frac{\Delta T}{{T - \tau }}} \right)^{{N_{X} }} } \right)^{K} \left( {\left( {1 - \frac{\Delta T}{{T - \tau }}} \right)^{{N_{X} }} } \right)^{{N_{Y} - K}}$$

where $\Delta T$ is the temporal tolerance and $\tau$ is the time lag between $X$ and $Y$. Significance test for coincidence measure is based on the null hypothesis that the number of coincidences can be explained by two independent series of randomly distributed events. The $p$-value of empirically observed number of coincidences ${K}_{e}$ with respect to the test distribution in Eq. (4) i.e. the probability to obtain a number of coincidences $K$ equal to or greater than ${K}_{e}$ is given by $P\left(K\ge {K}_{e}\right)=\sum_{{K}{\prime}={K}_{e}}^{{N}_{Y}}P({K}{\prime};{N}_{Y},1-{(1-p)}^{{N}_{X}})$. Null hypothesis is rejected if the $p$-value is smaller than the defined confidence level α.

### Supplementary Information

Supplementary Information.

## Data Availability

India-Water Resources Information System (India-WRIS) daily streamflow data used in this study is available at https://indiawris.gov.in/wris/#/RiverMonitoring. India Meteorological Department (IMD) provides daily high resolution gridded rainfall data can be accessed from https://www.imdpune.gov.in/cmpg/Griddata/Rainfall_25_NetCDF.html. European Space Agency Climate Change Initiative (ESA CCI) soil moisture dataset with daily temporal and 0.25° spatial resolution is available at https://esa-soilmoisture-cci.org/data. Global Aridity Index is available at 10.6084/m9.figshare.7504448.v5. Shuttle Radar Topographic Mission (SRTM) at 30 m spatial resolution is available at https://srtm.csi.cgiar.org/srtmdata/.
