# Supplementary material for: Baseflow significantly contributes to river floods in Peninsular India
Source: Sci Rep. 2024 Jan 13;14:1251. doi: 10.1038/s41598-024-51850-w (PMC10787776; doi:10.1038/s41598-024-51850-w)
Supplement: Supplementary file 1 — Supplementary Information. [file 41598_2024_51850_MOESM1_ESM.pdf]

Supplementary Information for:

Baseflow significantly contributes to river floods in Peninsular India

Shailza Sharma[1*], P. P. Mujumdar[1,2]

[1]Department of Civil Engineering, Indian Institute of Science, Bangalore, 560012, India.

[2]Interdisciplinary Centre for Water Research, Indian Institute of Science, Bangalore, India.

[*]shailzas@iisc.ac.in

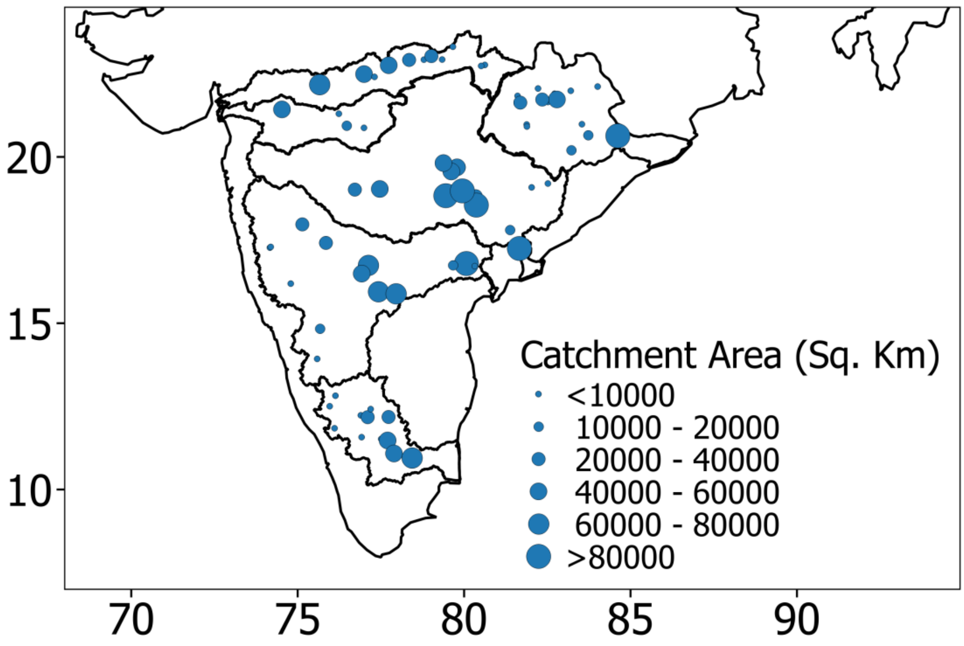

Figure S1. Locations of streamflow gauges with catchment areas in six major river basins of Peninsular India. The map is prepared in QGIS (Version 2.14.0 ‘Essen’ (2016), URL: http://qgis.org).

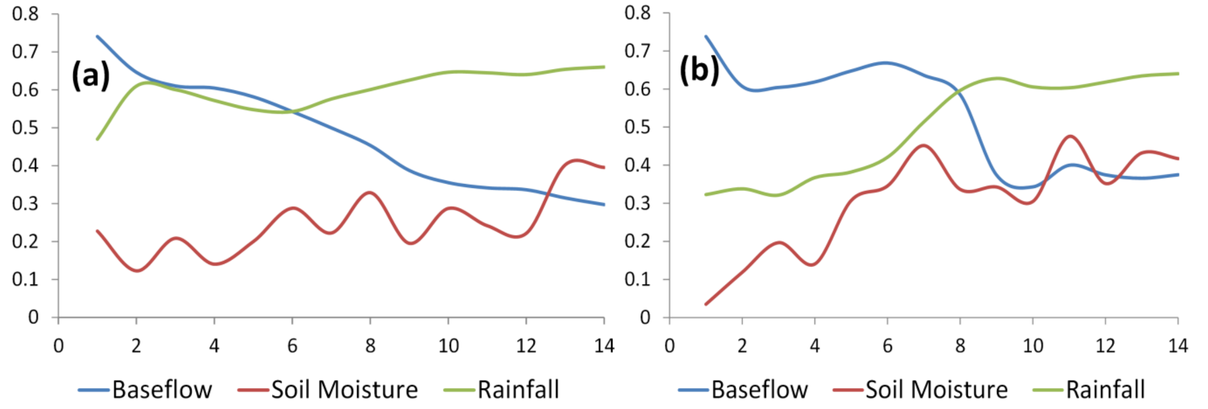

Figure S2. Correlation between flood magnitude and flood drives (baseflow, rainfall and soil moisture) for a range of antecedent periods (1-14 days) in Perur and G. R. Bridge catchments of Godavari river basins. Baseflow has a stronger and longer association with flood magnitudes 5-7 days). The plots are generated using R (Version 4.2.2 (2022), URL: https://www.R-project.org/).

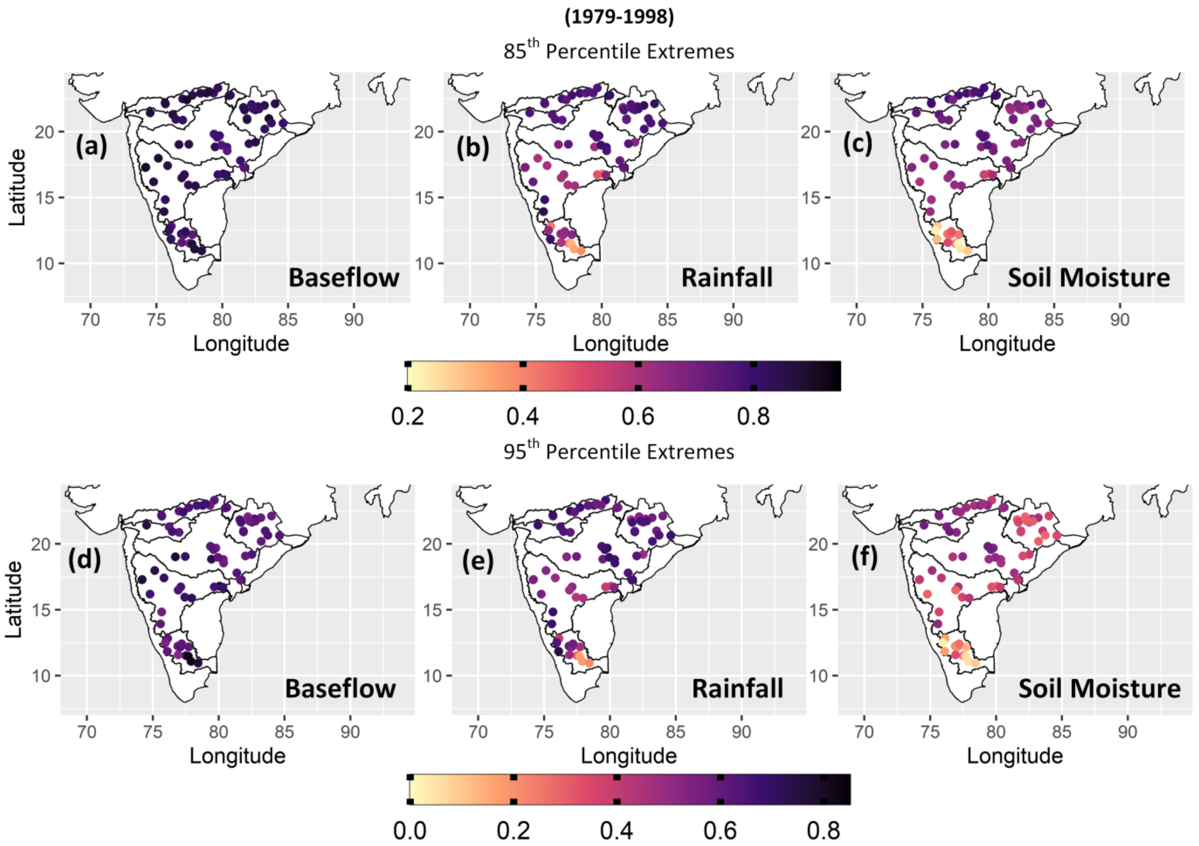

Figure S3. Trigger coincidence rates for 70 catchments in Peninsular India for the period 1979-1998. First row and second row presents the coincidence rates for pairs of flood magnitudes and flood drivers defined above 85th percentile and 95th percentile threshold, respectively. Baseflow is the dominant driver of floods irrespective of the flood magnitude. The maps are prepared in R (Version 4.2.2 (2022), URL: https://www.R-project.org/).

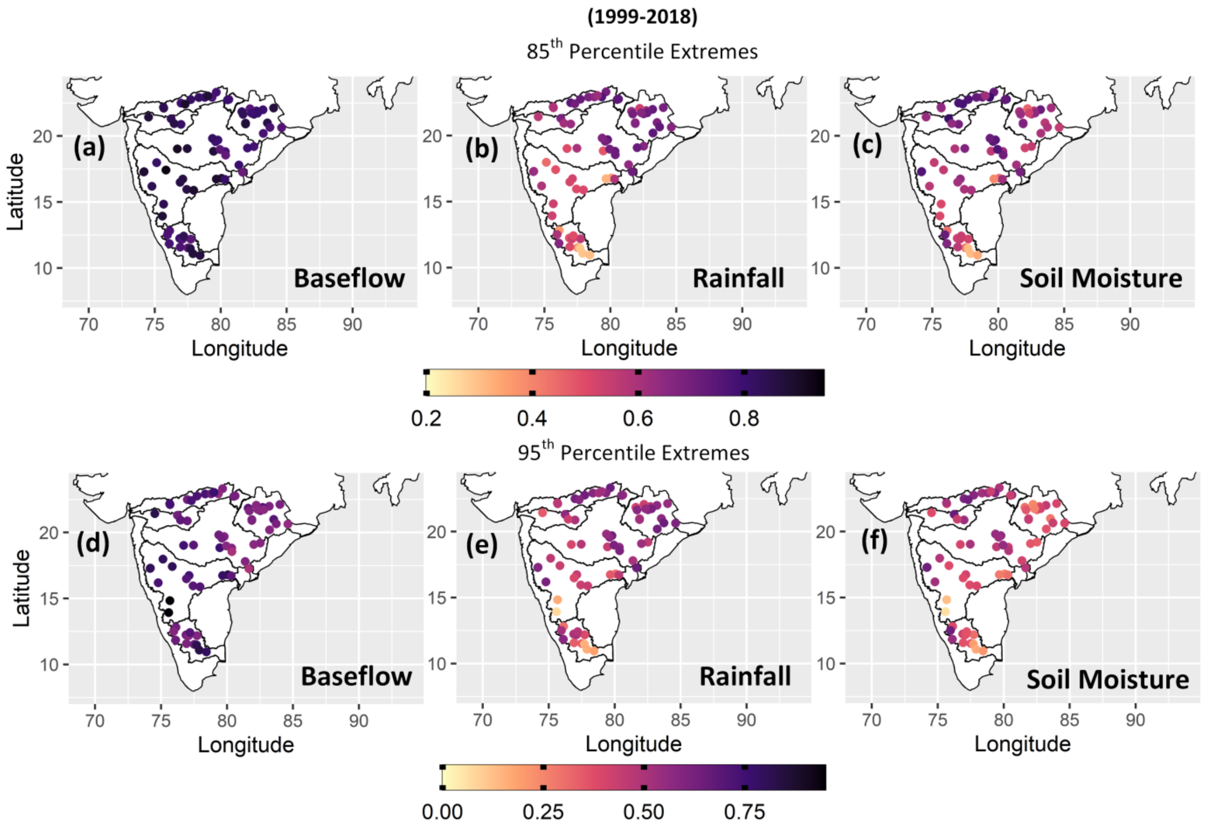

Figure S4. Trigger coincidence rates for Peninsular catchments for the period 1999-2018. Baseflow shows the highest triggering effect and soil moisture shows the lowest triggering effect on floods.

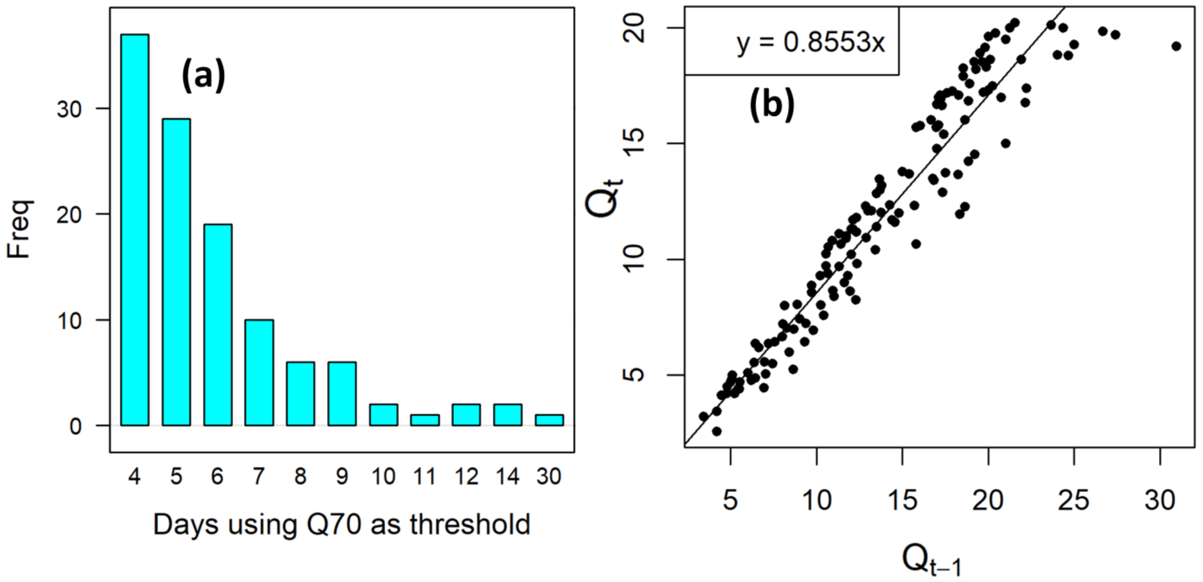

Figure S5. (a) Segment length and **(b)** Master Recession Curve (MRC) to estimate the recession constant at Haralahalli catchment of Krishna river basin.
